# Supplementary figures and images for: How array design creates SNP ascertainment bias
Source: PLoS One. 2021 Mar 30;16(3):e0245178. doi: 10.1371/journal.pone.0245178 (PMC8009414; doi:10.1371/journal.pone.0245178)

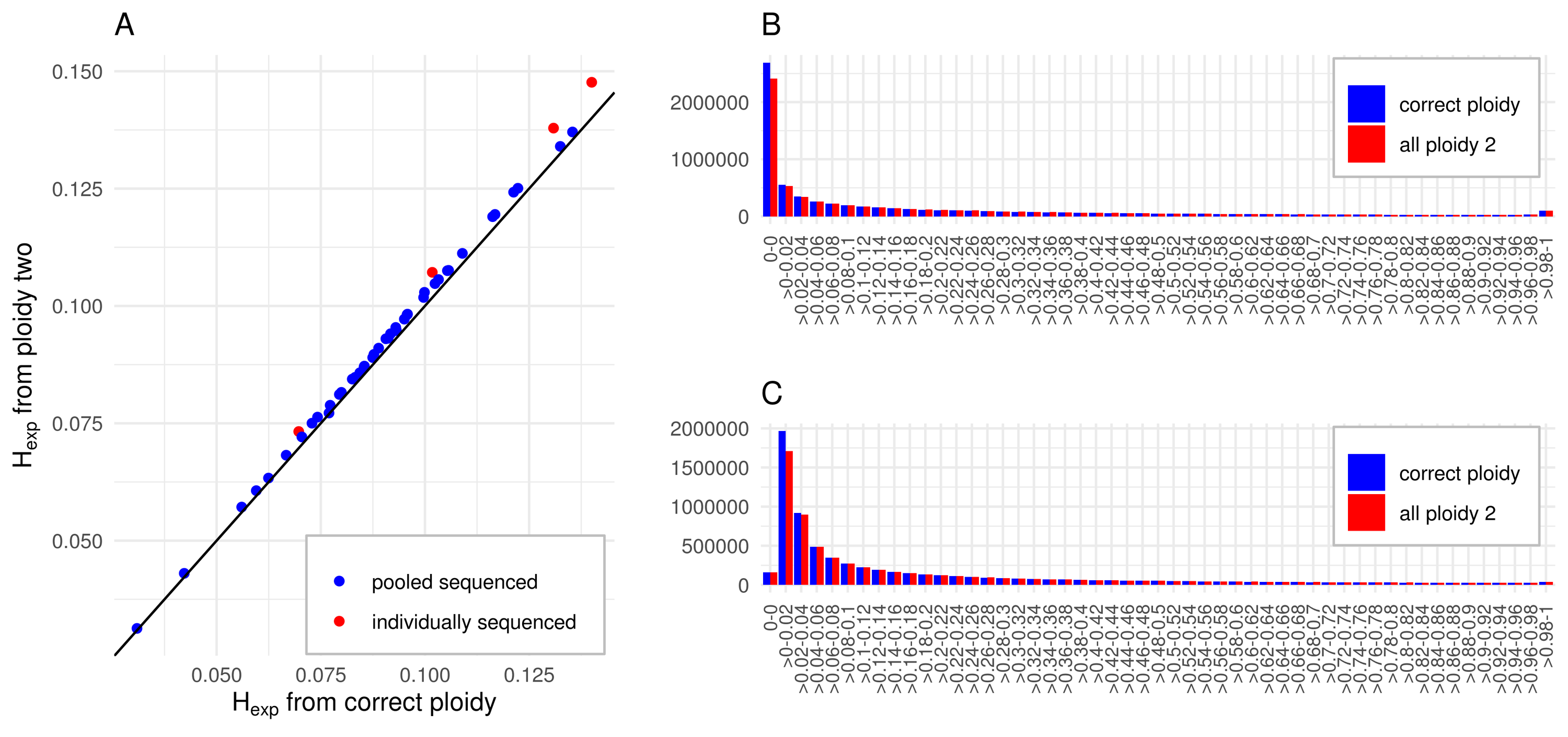

Supplement: S1 Fig — Expected Heterozygosity (Hexp) from the calling with assuming the correct ploidy vs. assuming ploidy two for all samples (A) and alternative allele frequency distributions of called alleles for individually sequenced (B) and pooled sequenced (C) populations. (TIF) [file pone.0245178.s005.tif]

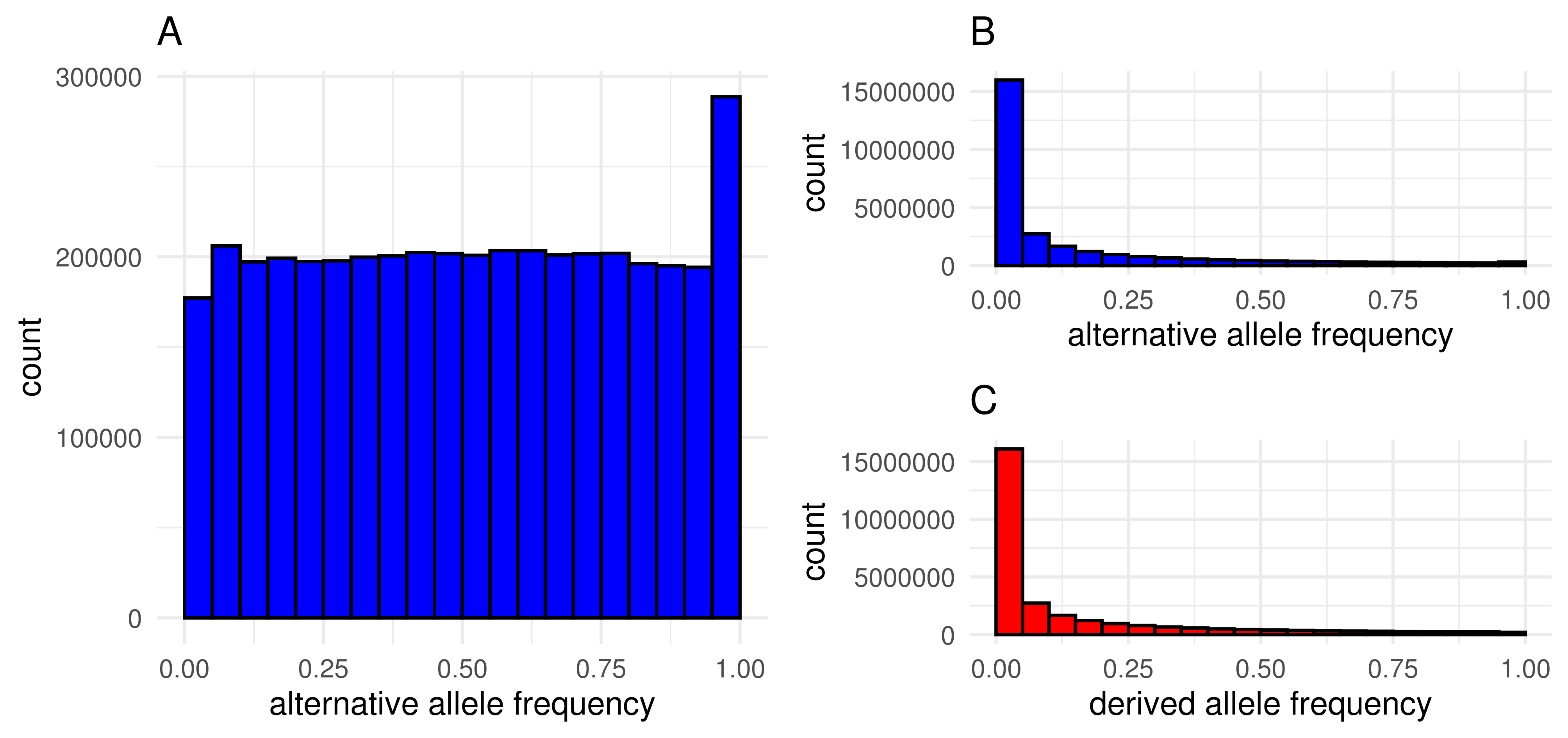

Supplement: S2 Fig — (TIF) [file pone.0245178.s006.tif]

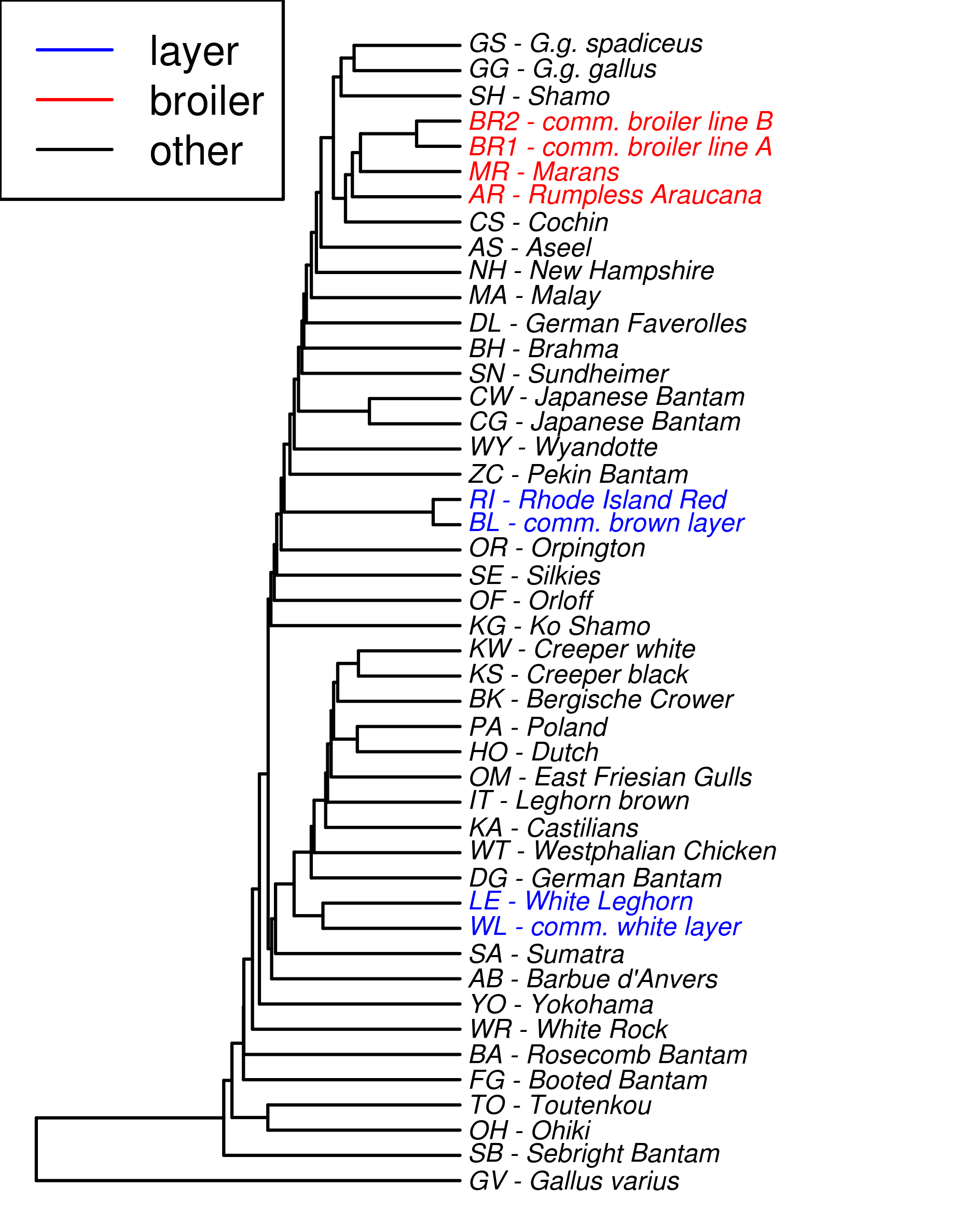

Supplement: S3 Fig — The tree was calculated from the filtered WGS SNPs. Populations defined as layers or broilers, which form in total the discovery set for the array design close to the original array, are highlighted. The plot was produced using the R package ape [55]. Note that the plot is only supposed to reveal close clustering chicken populations and cannot be interpreted in depth as chickens show a rich history of hybridization events. The interested reader can find all underlying pairwise Nei’s standard genetic distances [53] in S3 File and additionally pairwise FST values [54] in S4 File. (TIF) [file pone.0245178.s007.tif]

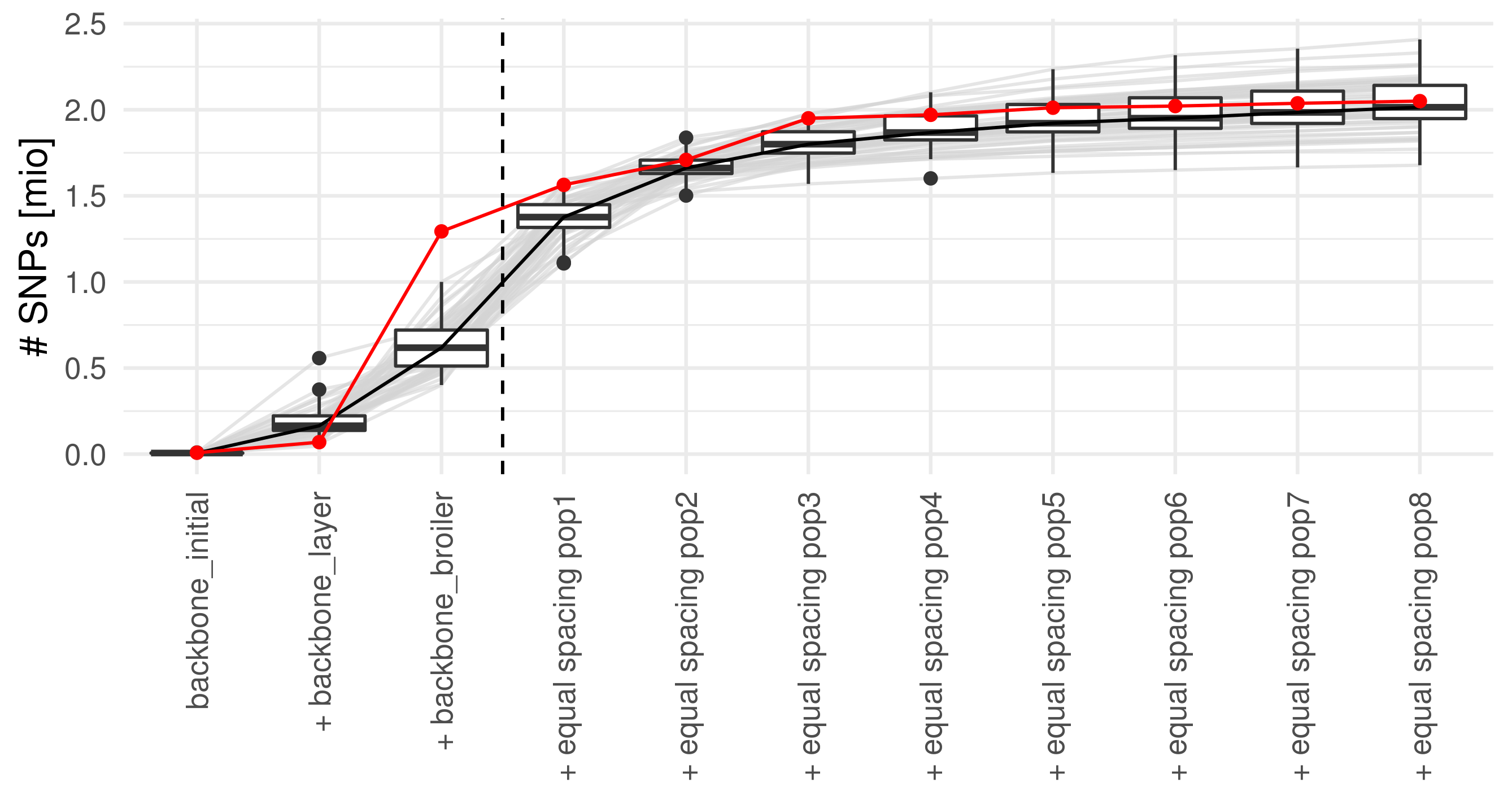

Supplement: S4 Fig — The red line and points represent the first remodeling according to the original array [21], while the dashed lines and the boxplots represent the 50 random population groupings and the black line the according median values. The algorithm starts with a very basic initial backbone and then adds SNPs to the backbone which are variable in either all layer lines or all broiler lines. Separated by a vertical line, the second part of the algorithm successively fills up potential gaps to achieve an equidistant coverage of 667 segregating SNPs/cM for each discovery population. (TIF) [file pone.0245178.s008.tif]

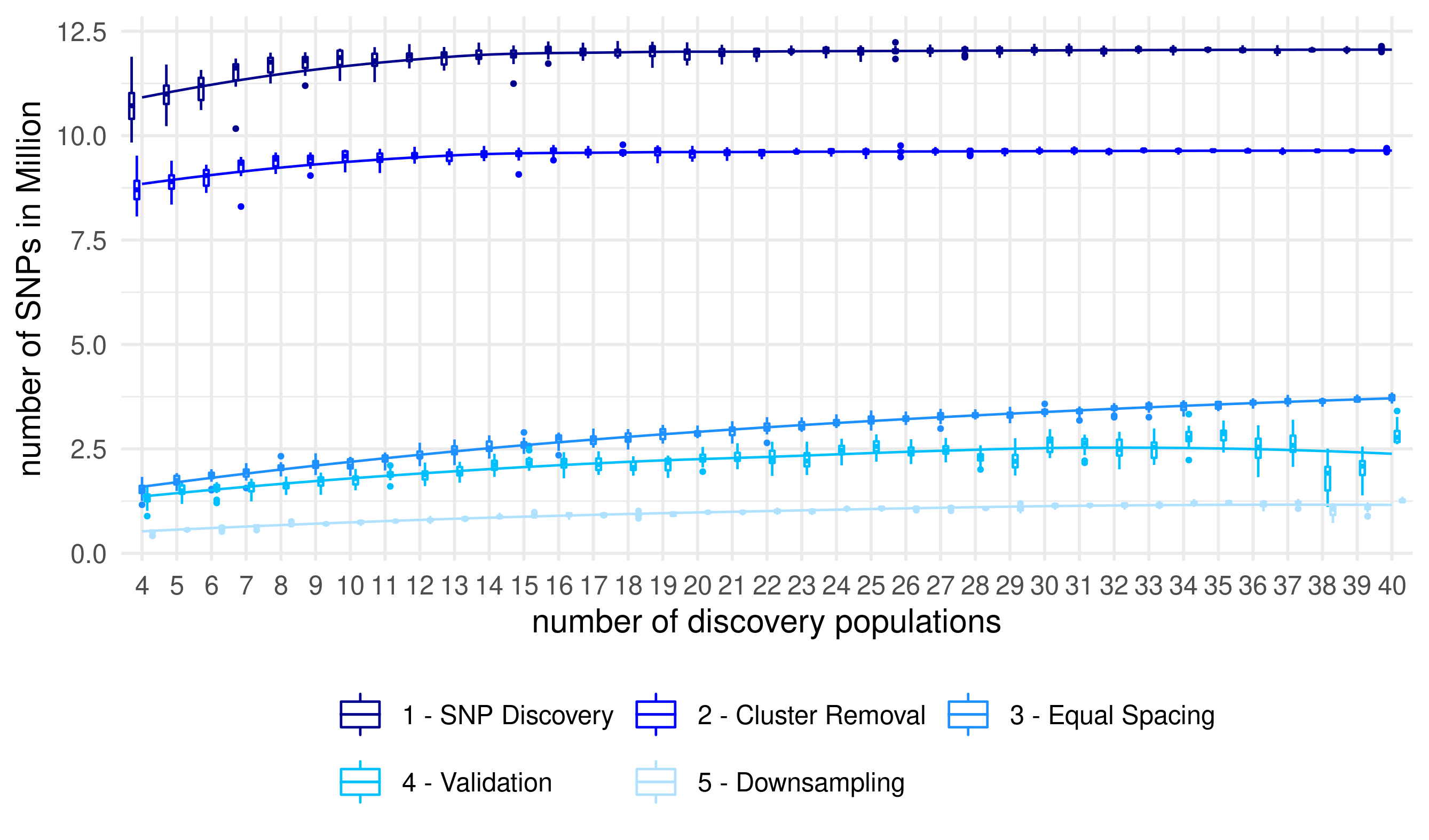

Supplement: S5 Fig — (TIF) [file pone.0245178.s009.tif]

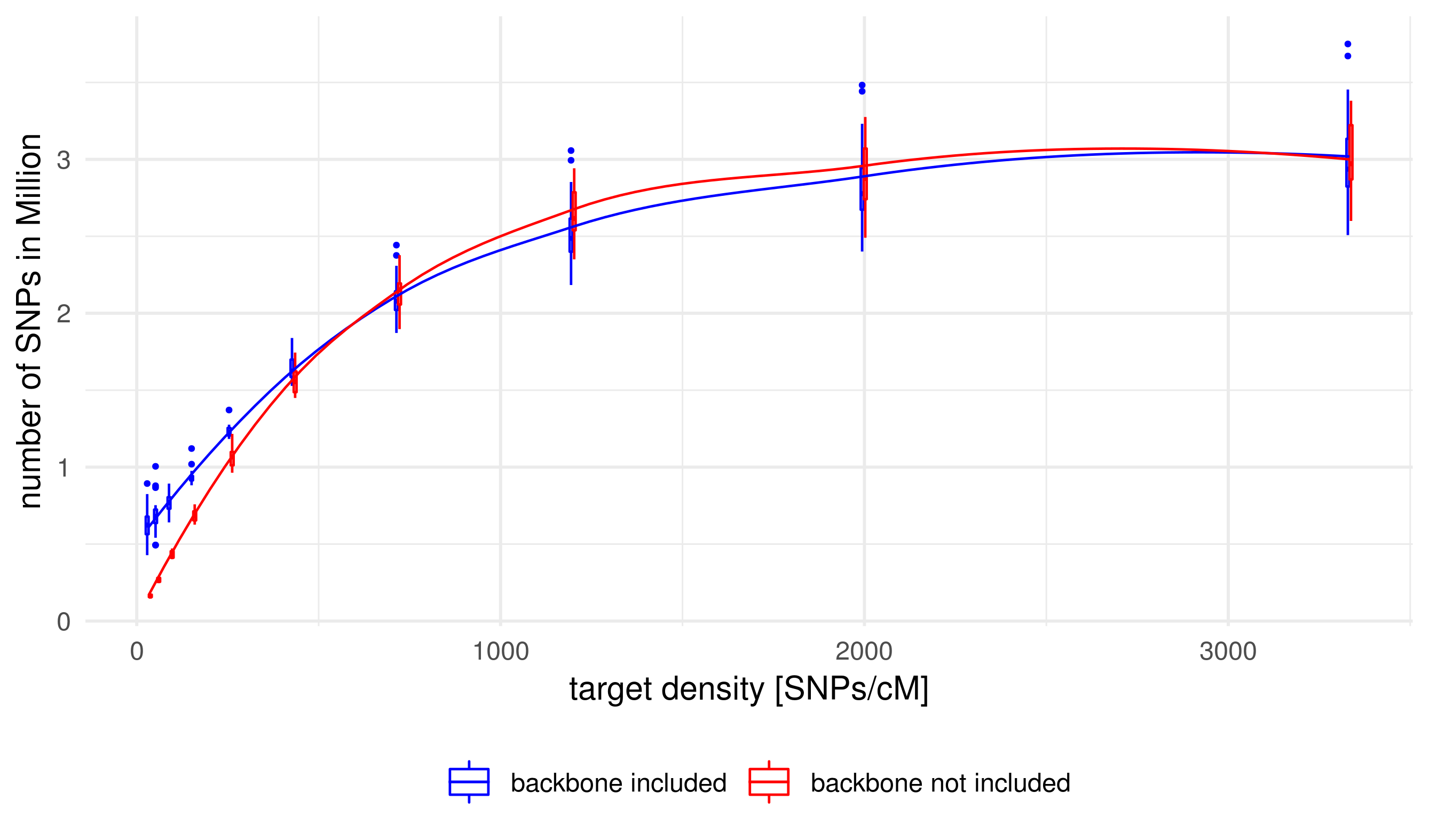

Supplement: S6 Fig — (TIF) [file pone.0245178.s010.tif]

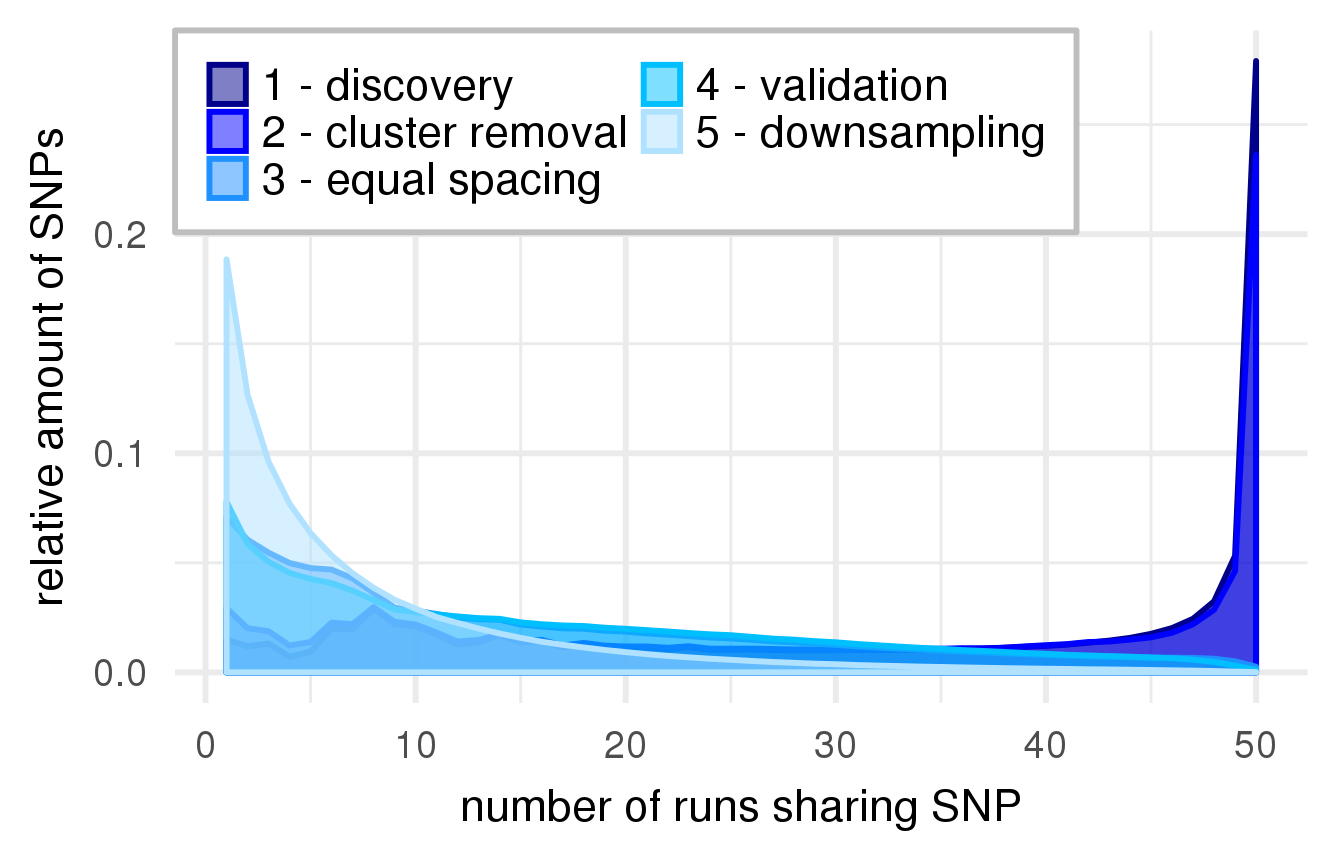

Supplement: S7 Fig — (TIF) [file pone.0245178.s011.tif]

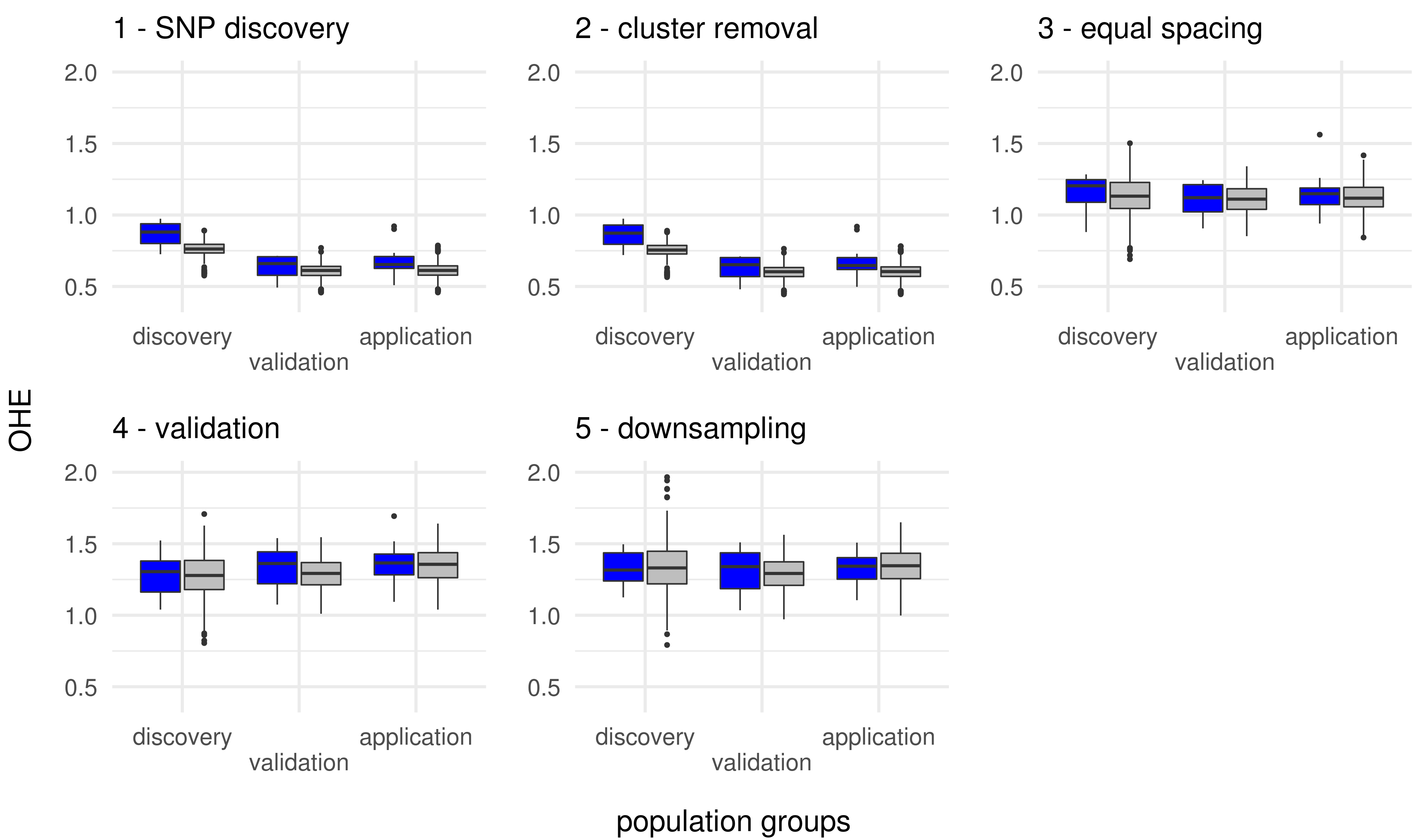

Supplement: S8 Fig — Discovery populations are chosen to represent populations which are comparable to the original array (blue) or 50 times random sampled (grey). (TIF) [file pone.0245178.s012.tif]

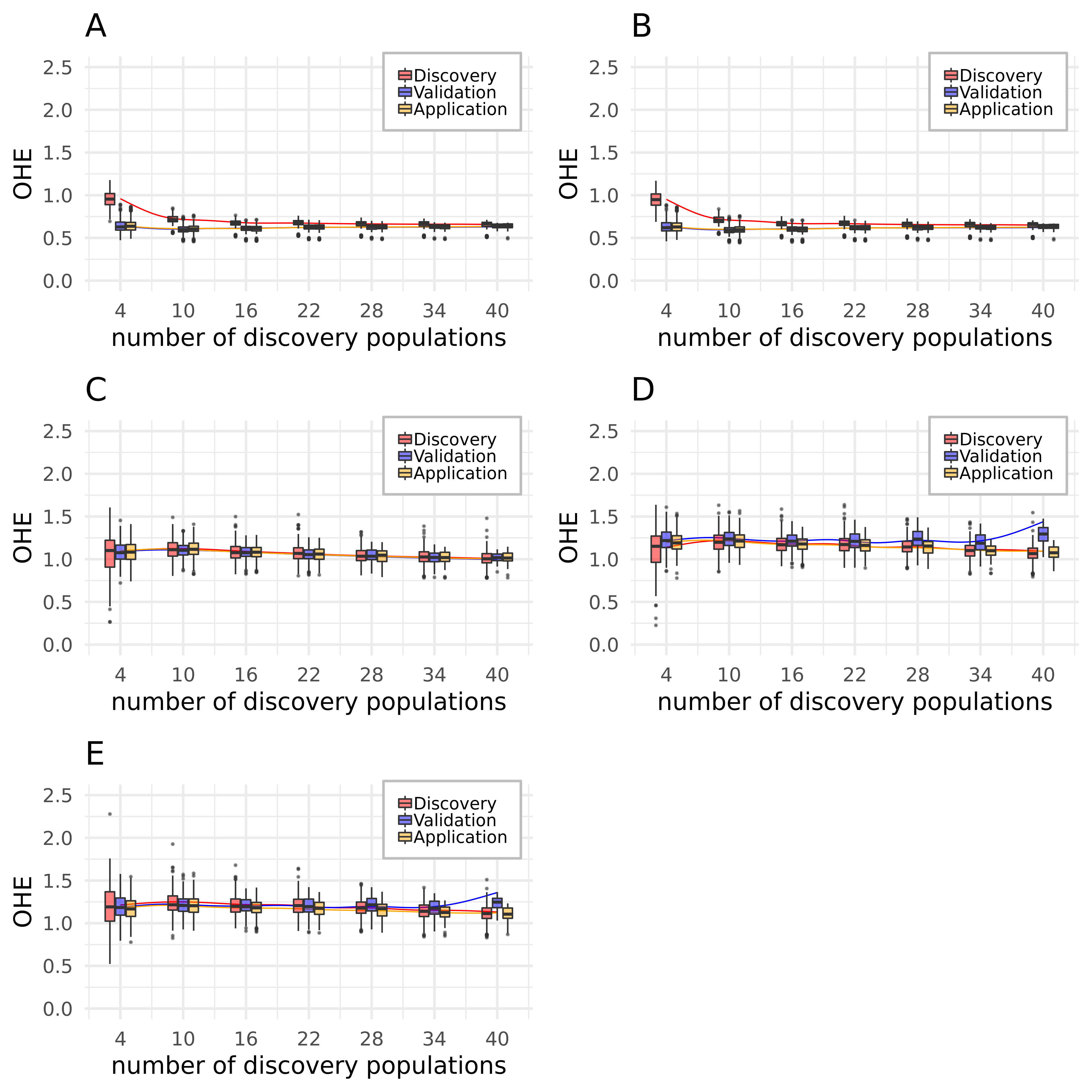

Supplement: S9 Fig — A—discovery, B–cluster removal, C–equal spacing, D–validation, E–downsampling. The Boxplots are only shown for a subset of the number of discovery populations, while the smoothing lines, which show the trend, are calculated from all observations. (TIF) [file pone.0245178.s013.tif]
